# Supplementary material for: Upconversion Emission Studies in Er3+/Yb3+ Doped/Co-Doped NaGdF4 Phosphor Particles for Intense Cathodoluminescence and Wide Temperature-Sensing Applications
Source: Materials (Basel). 2022 Sep 21;15(19):6563. doi: 10.3390/ma15196563 (PMC9570846; doi:10.3390/ma15196563)
Supplement: Supplementary file 1 [file materials-15-06563-s001.zip › materials-1856473-supplementary.pdf]

Supporting information

Upconversion emission studies in Er^{3+}/Yb^{3+} doped/codoped $NaGdF_4$ phosphor particles for intense Cathodoluminescence and wide range temperature sensing applications

A. Kumar[1],* H. Couto [2] and Joaquim C.G. Esteves da Silva [1]

[1] Research Centre in Chemistry (CIQUP), Departamento de Geociências, Ambiente e Ordenamento do Território, Faculdade de Ciências, Universidade do Porto, rua do Campo Alegre s/n, 4169–007 Porto, Portugal

[2] Instituto de Ciências da Terra – Pólo Porto, Departamento de Geociências, Ambiente e Ordenamento do Território, Faculdade de Ciências, Universidade do Porto, rua do Campo Alegre s/n, 4169–007 Porto, Portugal

* Correspondence: hcouto@fc.up.pt

Table S1: 980nm Pump power excitation dependent CIE colour co-ordinates and colour purities

S. No.	Pump Power (mW)	CIE-X	CIE-Y	Purity (%)
1	100	0.3381	0.3352	2.0
2	300	0.3604	0.3643	17.5
3	400	0.3640	0.3766	22.2
4	600	0.3683	0.4101	33.6
5	800	0.3636	0.4405	41.4
6	1000	0.3562	0.4607	45.4
7	1200	0.3458	0.4804	48.3
8	1400	0.3334	0.4993	50.4
9	1600	0.3268	0.5080	51.1
10	1800	0.3211	0.5189	52.8
11	2000	0.3146	0.5279	53.8
12	2200	0.3149	0.5286	54.0
